# Advocating for Change: Unveiling the Challenges of an Ultrasound-First Approach in Uncomplicated Renal Colic

**DOI:** 10.7759/cureus.88581

**Published:** 2025-07-23

**Authors:** Disha Bhargava, Rebecca Loney, Matthew Flannigan, Jeffrey Jones

**Affiliations:** 1 Department of Medicine, Michigan State University College of Human Medicine, Grand Rapids, USA; 2 Department of Emergency Medicine, Michigan State University College of Human Medicine, Grand Rapids, USA

**Keywords:** clinical algorithm, computer topography, healthcare utilization, imaging, radiation exposure, renal colic, ultrasound

## Abstract

Background: In recent years, medical literature has advocated for an ultrasound-first (US-first) approach for the diagnosis of uncomplicated renal colic. Despite these recommendations, computed tomography (CT) continues to be overutilized.

Objective: This project had a three-pronged approach: 1) to investigate the current diagnostic choice and factors that influence it for uncomplicated renal colic within our local healthcare system, 2) to create and implement an algorithm to emphasize the use of a US-first approach for uncomplicated patients, and 3) to identify the effectiveness of the clinical algorithm.

Methods: An initial five-month retrospective evaluation (2020-2021), including regional and tertiary emergency departments (EDs), was performed to review emergency provider practice patterns when investigating renal colic. In conjunction with this, a clinician survey was performed to identify factors affecting providers' choice of diagnostic imaging for renal colic. Next, a multidisciplinary group of providers developed a clinical algorithm advocating for a US-first approach to diagnosing uncomplicated renal colic. Finally, a postintervention retrospective evaluation was conducted in 2023 to evaluate the effectiveness of this clinical algorithm.

Results: The initial retrospective study showed CT as the primary diagnostic choice (used in 102 out of 135 patients) for investigating uncomplicated renal colic. After the implementation of the clinical algorithm, there was a significant reduction in CT scans by 14.2% (p = 0.016). There were no significant differences in the need for urological interventions, hospitalization, repeat ED visits within one week, or missed serious diagnoses.

Conclusions: This study demonstrated the effectiveness of a multidisciplinary-derived clinical algorithm in successfully reducing the use of CT scans in uncomplicated renal colic. The US-first approach was utilized in a higher percentage of patients following the introduction of the clinical algorithm; however, CT still dominated as the primary diagnostic choice.

## Introduction

Of the two million annual emergency department (ED) visits for renal colic, 90% undergo computed tomography (CT) imaging [1], even though research indicates that imaging for renal colic in the ED seldom changes immediate management [2]. Renal colic has a 5%-15% lifetime incidence and is typically a self-limited condition, with most stones passing spontaneously [3]. The widespread use of CT has demonstrated no discernible improvement in patient-centered outcomes such as admissions, urologic interventions, or the proportion of patients diagnosed with kidney stones over the last two decades [4].

In the late 1980s, ultrasound (US) was recognized to have similar test characteristics to an intravenous pyelogram, the previously recognized gold standard test for renal colic [5]. However, a decade later, the prevalence of CT scanning technology increased, and its superior sensitivity and specificity for renal colic were identified. Subsequently, multiple publications highlighted the additional benefit of CT in identifying both the size and location of the stone, which provides a better assessment for those patients who may require intervention. These studies also highlighted the additional benefit of using CT to avoid missing a serious alternative diagnosis [6,7]. As a result, CT was established as the new gold standard for the investigation of renal colic.

As healthcare costs continue to rise and awareness of radiation exposure risk grows, recent studies have advocated for an ultrasound-first (US-first) approach for patients with uncomplicated renal colic [2]. This approach is supported by the Choosing Wisely Campaign, which has been designed to reduce unnecessary healthcare costs. Within this campaign, American College of Emergency Physicians (ACEP) guidelines support "Avoid ordering CT of the abdomen and pelvis in young otherwise healthy ED patients (age <50) with known histories of kidney stones or ureterolithiasis, presenting with symptoms consistent with uncomplicated renal colic" [8].

Utilization of an algorithm that adopts a US-first approach may avoid ionizing radiation in up to 70% of patients [2,9]. In uncomplicated patients with a high pretest probability for renal colic, a US exam can be used in combination with other clinical findings as the only diagnostic imaging method [10]. This research aims to identify the current approach and evaluate factors underlying providers' decisions for imaging choice in renal colic, create and implement a clinical algorithm with a US-first approach for uncomplicated renal colic, and identify the effectiveness of the clinical algorithm.

## Materials and methods

Data collection

This study was approved by the Institutional Review Board for each institution as a quality improvement project. The project consisted of a three-pronged approach. First, an initial evaluation of diagnostic studies used in renal colic (baseline) was completed in conjunction with a clinician survey to assess factors underlying provider choice. Second, a clinical algorithm was created by a multidisciplinary team. Finally, following the implementation of the clinical algorithm, a postintervention evaluation of the diagnostic approach chosen for patients with suspected uncomplicated renal colic was conducted.

Part 1: preintervention data collection and survey

The initial retrospective study was performed between December 2020 and May 2021 to determine the baseline emergency provider (EP) diagnostic preferences for patients with uncomplicated presentations of suspected renal colic at two regional (approximately 25,000-30,000 each/year) and one tertiary care (110,000 patients/year) EDs. Data collected included key demographics, clinical features, diagnostic tools, and treatment outcomes. Patients 18-50 years old were identified on EPIC (Epic Systems Corporation, Verona, WI) based on a chief complaint of flank pain or suspected ureteral stone or final diagnosis of renal colic (International Classification of Diseases, Tenth Revision, Codes: N20.0, N20.1, and N23). All identified patients during the time period were extracted into a REDCap database (Research Electronic Data Capture, Vanderbilt University, Nashville, TN). Each unique patient presentation was reviewed with data extracted manually from EPIC and entered into the REDCap database.

**Figure 1:**
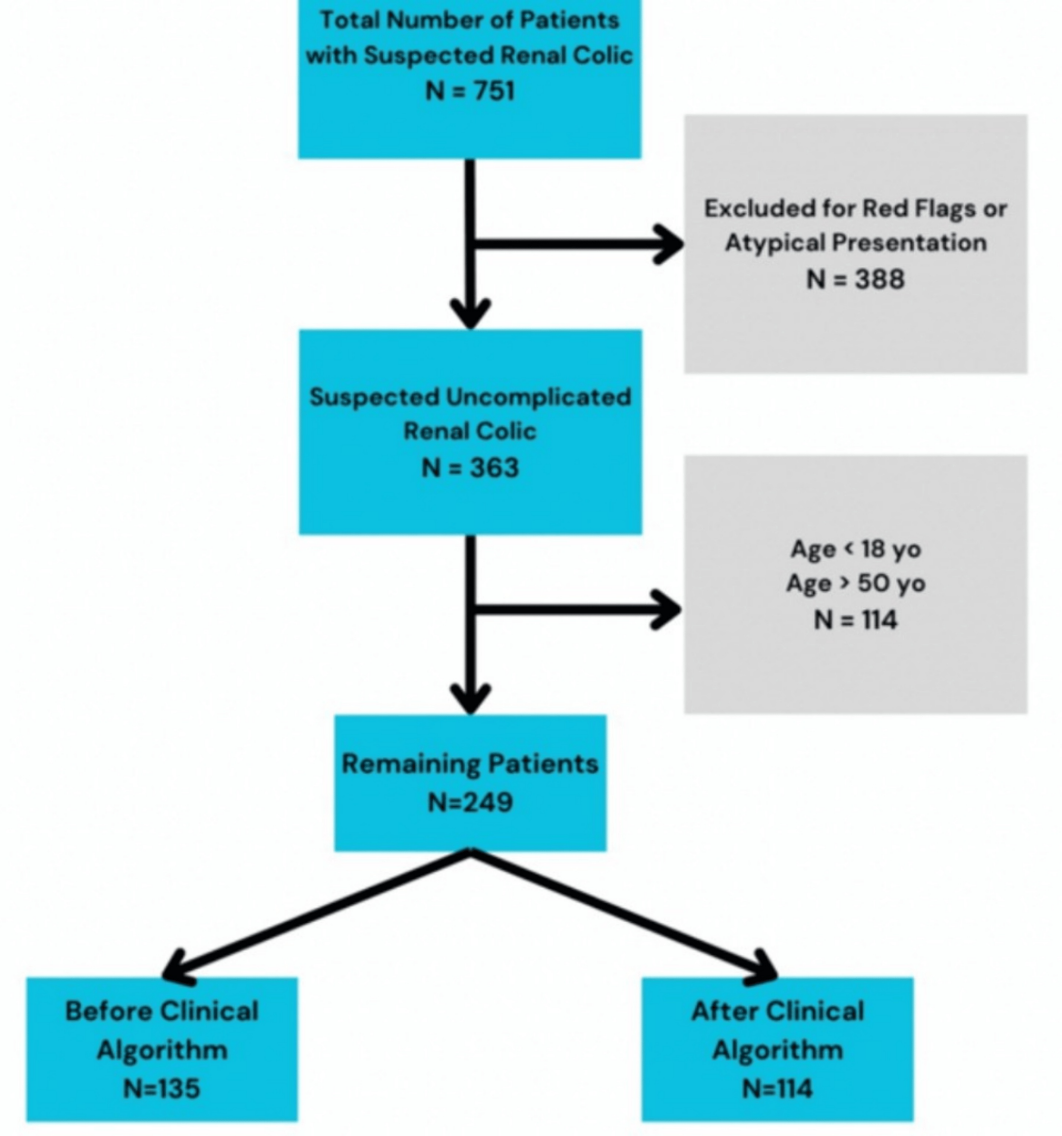
Workflow of the total number of patients from initial data extraction to final number of patients included in the study

Patients were excluded for the following criteria: morbid obesity (men >130 kg; women >115kg), concern for a more serious alternate diagnosis, or if the patient had a single/transplanted kidney. Concern for a more serious alternative diagnosis was determined from key elements in the treating provider’s medical decision-making section and/or red flags from the chart review. Red flags included signs of systemic infection, abnormal physical exam findings, or abnormal urinalysis. The data extraction team included medical students and emergency physicians, including the primary investigator. For any patients with unclear charting, the Principal Investigator reviewed these independently to ensure they met the appropriate inclusion criteria before they were included in the study population. Figure 1 shows the workflow of patients that were included at initial extraction to the final number of patients included in the pre- and postintervention data collection.

Additionally, an online survey was sent to 250 EPs employed at the study sites. Recipients were sent a link to the survey using Microsoft Forms (Microsoft Corporation, Redmond, WA) and were sent a reminder email to participate two weeks later. The survey collected background data on provider demographics, experience with the interpretation of US images, and perceived barriers to using a US-first approach in the evaluation of renal colic. Then, the survey asked providers to select the most appropriate diagnostic imaging choice for nine structured clinical scenarios. This included no imaging, point-of-care ultrasound (POCUS), radiology performed ultrasound (RADUS), or CT. These were selected from a recent multispecialty consensus publication by Moore et al. in 2019 that had Excellent (8/9) or Perfect (9/9) agreement among a multidisciplinary group of three emergency physicians, three radiologists, and three urologists [1].

Part 2: creation and implementation of the algorithm

**Figure 2:**
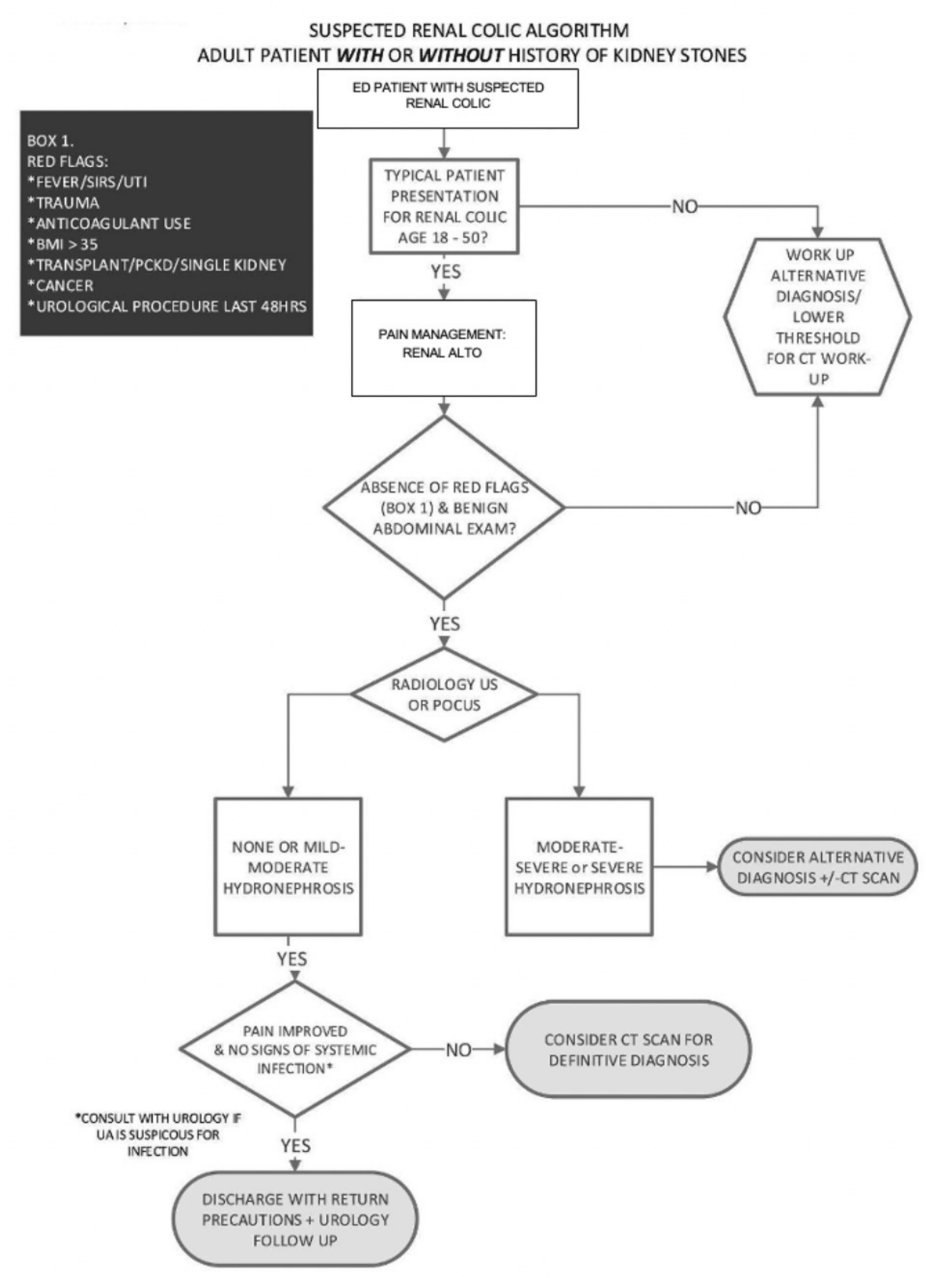
Multidisciplinary algorithm for patients presenting with renal colic SIRS: systemic inflammatory response syndrome; UTI: urinary tract infection; ALTO: alternative to opioids; BMI: body mass index; ED: emergency department; CT: computed tomography; US: ultrasound; POCUS: point-of-care ultrasound

After reviewing the results of part one, we identified key stakeholders and developed a working group to create a clinical algorithm to advocate for a US-first approach for adult patients 18-50 years old with an uncomplicated renal colic presentation (Figure 2).

This local multidisciplinary group of physicians included one urologist, one radiologist, and two emergency physicians. The need for a local algorithm, which was based on the National Algorithm from the “Choosing Wisely campaign,” was identified based on the lack of current adoption of national guidelines as well as provider perceived value in a collaborative, multidisciplinary approach [8]. Collaboration between specialties was considered vital so that all parties involved in the assessment and management of these patients were in agreement with a US-first approach for low-risk patients.

An initial introduction to this new clinical algorithm was provided through departmental meetings, resident didactics, and on-shift education. The algorithm was also posted in the department and made available to EPs through the health system’s intranet.

Part 3: postintervention data collection

Finally, a postintervention evaluation was completed, using the same criteria as the initial data collection, between December 2022 and March 2023 to evaluate the effectiveness of the clinical algorithm for leveraging a US-first approach and reducing CT utilization for uncomplicated renal colic.

Clinical data management included the following: 1) data management and 2) a limited patient data set was stored on the Research Electronic Data Capture (REDCap) database, Vanderbilt University, TN. Data collection was obtained through an EPIC electronic medical record chart review. These data were reviewed by the Principal Investigator during data collection and at the end to ensure quality control and reduce any interobserver variations with data collection or errors with data transcription.

Statistical analysis

Categorical variables were described using percentages. Continuous variables were described using medians and interquartile ranges. Comparison in categorical data and numerical data was done with chi-square or Fisher's exact test and T-test or Wilcoxon rank, respectively, for patient factors, imaging modality used, and need for interventions. Sample size determinations were based on a study from Raja et al. that indicated a required total of 248 patients to be powered for significant outcomes [11].

## Results

There were 135 patients included in our baseline determination of imaging selected in the evaluation of renal colic, and 114 patients included in the post-clinical-algorithm implementation measurement of imaging practices, for a total of 249. Patient characteristics in the pre- and postalgorithm groups are presented in Table 1. This sample demonstrates that there are no significant differences in patient age, sex, ethnicity, or weight.

**Table 1:** Patient demographics ^*^Fisher's exact test IQR: interquartile range

Characteristic	Postalgorithm (n = 114)	Prealgorithm (n = 135)	p value
Hospital location, n (%)			
A	53 (46.49%)	44 (32.59%)	<0.001^*^
B	41 (35.96%)	40 (29.63%)	
C	16 (14.04%)	49 (36.30%)	
Other	4 (3.51%)	2 (1.48%)	
Patient age, years, median (IQR)	36.00 (29.00-42.75)	36.00 (28.50-44.00)	0.904
Patient gender, n (%)			
Male	56 (49.12%)	61 (45.19%)	0.610
Female	58 (50.88%)	74 (54.81%)	
Patient ethnicity, n (%)			
Non-Hispanic	108 (95.58%)	124 (95.38%)	0.943
Hispanic	5 (2.9%)	6 (3.8%)	
Weight (kg), median (IQR)	86.45 (68.36-98.25)	84.82 (67.17-98.00)	0.745

There were no significant differences in the morphine milligram equivalent used between the pre- and postintervention groups (Table 2).

**Table 2:** Descriptive statistics of the ED visit ^*^Wilcoxon rank-sum test IQR: interquartile range; ED: emergency department

Characteristic	Postalgorithm (n = 114)	Prealgorithm (n = 135)	p value
Morphine equivalents, median (IQR)	5 (4-10)	4 (2.54-7.75)	0.131
Time to disposition (hours), median (IQR)	3.30 (2.70-4.10)	2.87 (2.10-3.94)	0.003
Serious alternative diagnosis, n (%)	0 (0%)	2 (1.49%)	>0.999^*^
Need for urologic intervention, n (%)	17 (14.91%)	17 (12.69%)	0.612
Need for hospitalization, n (%)	19 (16.67%)	17 (12.59%)	0.362
Repeat ED visit within one week from index presentation, n (%)	12 (10.53%)	13 (9.70%)	0.830

There was also no significant difference in serious alternative diagnosis, need for urologic intervention, or need for hospitalization. There was a significant increase, however, in time to disposition from 2.87 to 3.30 hours from pre- to postintervention group. Chart abstractors noted two missed serious diagnoses included in the preintervention group. A dedicated chart review was performed on these two patients by the corresponding author, and it became clear that these patients were included in error. On both occasions, the treating provider’s medical decision-making indicated that they were considering a more serious diagnosis and, therefore, were not appropriate for inclusion.

Imaging modality (Table 3) shows a significant decrease in CT utilization in the postimplementation group from 75.56% to 61.40% or an absolute reduction of 14.16%. There was also a significant increase in patients who received POCUS from 3.70% to 16.67% between pre- and postintervention, indicating a significant increase in POCUS performance.

**Table 3:** The number of patients per imaging method used before and after implementation of the clinical tool CT: computed tomography; POCUS: point-of-care ultrasound

Characteristic	Postalgorithm (n = 114)	Prealgorithm (n = 135)	p value
CT scan	70 (61.40%)	102 (75.56%)	0.016
Ultrasound only	28 (24.56%)	25 (18.52%)	0.246
CT scan only	57 (50%)	94 (69.63%)	0.002
CT scan and ultrasound	13 (11.40%)	8 (5.93%)	0.121
Image type: POCUS	19 (16.67%)	5 (3.70%)	<0.001

From the survey, there was a 45% (112/250) response rate from clinicians who work at a combination of both regional and academic centers. Of the respondents, 54% were attending physicians, 33% advanced practice providers, and 13% Emergency Medicine residents. Even though 60% of respondents reported having basic to advanced US training (physicians), the majority (88%) reported “rarely” or “never” performing POCUS to investigate for renal colic. Overall, 59% of them perceived “significant” barriers to implementation of a US-first approach. Significant barriers to implementing a US-first approach included concerns about missing a more serious diagnosis, lack of US availability, and a lack of treatment algorithms and knowledge to implement them. The nine clinical scenarios showed a poor understanding of American Board of Emergency Medicine's “Choosing wisely" campaign and ACEP’s Emergency Quality Network E-Qual “Consensus for imaging in renal colic.”

## Discussion

Despite concerns regarding cost and cumulative radiation exposure, the “CT-first” approach for diagnosis of renal colic continues to be the predominant choice in our region. This choice is driven by various factors, including perceived patient expectations for diagnostic certainty and “provider fears” of missing a serious alternate diagnosis [2,12]. Our initial retrospective review revealed that approximately 76% of patients presenting to the ED with renal colic received a CT scan. This is slightly lower than the CT rate found in a recent retrospective study of over 300,000 patient chart reviews [13]. While a CT-first approach is appropriate for complicated presentations, evidence continues to support a US-first approach for low-risk patients with the potential to reduce hospital costs and radiation exposure [2,5,14,15].

While there was a modest reduction in CT scans performed during the study period, it is projected that the US-first approach could be further utilized. One of the objectives of this study was to analyze factors that contributed to CT over US as the preferred diagnostic choice for uncomplicated renal colic. Our clinician survey revealed that 59% of physician respondents perceived multiple barriers to a US-first approach. Barriers included concerns about missed serious diagnoses, limited US availability, a lack of expertise with POCUS, a lack of consensus algorithm, and concerns about poor reimbursement. Some of the identified barriers are addressed below.

Concern for missed serious diagnosis

A study by Hastings and Powers [16] observed trends in diagnosis, demographics, and diagnostic test utilization in patients presenting to the ED with atraumatic abdominal pain. The overall conclusion demonstrated that abdominal pain has become time-, money-, and resource-intensive. Despite a threefold increase in the utilization of CT scans, there has been only a small impact on diagnostic specificity, and no change in admission rates or the number of seriously missed surgical illnesses. Even with this evidence and multiple additional studies that confirm the safety of a US-first approach in low-risk renal-colic patients, barriers remain for many providers [1,15,17]. It is believed that both cultural expectations for definitive answers (i.e., stone size and location) and a concern for the medicolegal ramifications of a missed diagnosis contribute significantly to this resistance to change diagnostic habits.

US availability/lack of expertise with POCUS

Only 28.13% (including US only: 18.52; CT and US: 5.93; POCUS: 3.7) of patients in the preclinical algorithm group underwent US as part of their diagnostic work-up, despite all patients fitting into the appropriate "low-risk" group. Lack of US availability may have impacted this; however, the US was available 24/7 at the tertiary care site. Likely, time delays for US technician availability and delays in US reads also impacted the providers' diagnostic choice.

The clinician survey and low utilization of POCUS by EPs in this study highlighted a lack of comfort in our local group of EPs to perform POCUS on patients with suspected renal colic. Recent studies have found EP to have moderate to high sensitivity for identifying hydronephrosis, serving as an indirect indicator of ureteral stones, when compared to emergency radiologist interpretation [10,14]. Following education and implementation of the algorithm, there was a clinically significant yet small (3.70%-16.67%; p < 0.001) increase in the use of POCUS by EPs. This result was encouraging as the focus of the study on education was aimed at raising awareness of the newly created algorithm and provided minimal emphasis on POCUS training. Providers might have felt more empowered to perform POCUS, knowing that this was an endorsed option by our local multidisciplinary team. Alternatively, they may have felt more comfortable after receiving a quick five-minute refresher regarding our department’s recommended views and documentation standards for this exam. It is speculated that the rate of POCUS utilization could continue to increase with a greater emphasis on provider training.

Increased POCUS utilization in the ED could help alleviate barriers, such as the availability of US technicians. US availability at regional hospitals during this study was limited due to single technician coverage on weekends and on-call coverage overnight between 1 and 7 a.m. This factor likely contributed to the longer length of stay (LOS) for patients in the post-clinical algorithm group. This study was not powered to assess LOS in the subset of patients that underwent POCUS by EPs. However, anecdotally, the immediate availability of POCUS results to the EP would directly correlate with a decrease in LOS for these patients. The frequent factor affecting disposition time is waiting for US results, not inadequate pain control.

Lack of a consensus algorithm

Many survey respondents felt that having a locally derived clinical algorithm would provide additional guidance in medical decision-making. This study validated this impression by demonstrating a 14.16% reduction in CT utilization after the implementation of a locally derived clinical algorithm.

A possible concern from EPs adopting a US-first approach could be an "unsaid" expectation that the Urology team would request a CT scan when consulting on these patients. Collaboration with providers in this group revealed that urologists in our region were in agreement with the campaign, and there was actually a request for another algorithm in patients with "known" current stone to help limit unnecessary imaging in these patients.

Given the prevalence of renal colic, even this modest reduction in CT scan utilization could have a significant impact on this patient group’s lifetime radiation exposure. When using a radiation risk calculator, a standard CT exam exposes a 40-year-old man to 8 mSv and results in future risk for cancer in one in 1,878 individuals [18]. Based on the annual incidence of ED presentations for renal colic in the United States at 1.2 million, a 14% reduction in CT imaging would theoretically result in 89 fewer persons developing cancer each year. It is important to recognize that the risk associated with radiation exposure is not linear; most renal colic patients have multiple recurrences, and therefore, this reduction in radiation exposure is likely significantly underestimated [2].

Limitations

Limitations of this study include the subjective nature of chart review, particularly in interpreting medical decision-making from provider notes, which do not always clearly outline the rationale behind diagnostic imaging choices. The initial retrospective review was conducted during the height of the COVID-19 pandemic and may have led to a reduction in POCUS utilization compared to radiology US imaging, primarily due to the stringent requirements for cleaning the bedside US between patients. Additionally, while there was a notable decrease in CT scans, the study also revealed a significant increase in the time-to-disposition in the post-clinical algorithm group. A longer LOS is likely attributed to the longer wait for the radiology-performed US compared to a CT scan report, or due to a subset of patients who underwent the US and then still required a CT scan.

A longer ED LOS is not insignificant and can contribute to ED overcrowding/boarding, which has been shown repeatedly to impact overall patient management negatively [19]. However, the increased radiation exposure for patients with renal colic, without a demonstrated change in patient management, has a series of unintended consequences that must be weighed. By increasing emergency provider POCUS utilization, there is an opportunity to reduce radiation exposure and healthcare costs while having minimal impact on departmental efficiency. This study was not powered to determine if patients undergoing POCUS instead of RADUS would have a shorter LOS; however, this is anecdotally suspected.

## Conclusions

This study demonstrates the effectiveness of creating a multidisciplinary clinical algorithm to reduce radiation exposure and imaging cost for the evaluation of uncomplicated renal colic in the ED. The US-first approach did increase ED LOS, but this could be decreased by either increasing ED POCUS utilization or radiology-performed US efficiency. It is crucial to acknowledge that despite advocacy for the US-first approach, substantial hesitation persists among EPs. The study's authors argue that this reluctance is deeply rooted in a healthcare culture that values efficiency, wrestles with litigation for missed diagnoses, and struggles with diagnostic uncertainty. Our findings emphasize the importance of addressing barriers to a US-first approach through advocacy efforts led by local emergency providers and fostering collaboration among multidisciplinary teams.

## References

[1] Moore CL, Carpenter CR, Heilbrun ME (2019). Imaging in suspected renal colic: systematic review of the literature and multispecialty consensus. J Am Coll Radiol.

[2] Nicolau C, Claudon M, Derchi LE (2015). Imaging patients with renal colic-consider ultrasound first. Insights Imaging.

[3] Patti L, Leslie SW (2024). Acute Renal Colic. https://pubmed.ncbi.nlm.nih.gov/28613743/.

[4] Westphalen AC, Hsia RY, Maselli JH, Wang R, Gonzales R (2011). Radiological imaging of patients with suspected urinary tract stones: national trends, diagnoses, and predictors. Acad Emerg Med.

[5] Sinclair D, Wilson S, Toi A, Greenspan L (1989). The evaluation of suspected renal colic: ultrasound scan versus excretory urography. Ann Emerg Med.

[6] Goldstone A, Bushnell A (2010). Does diagnosis change as a result of repeat renal colic computed tomography scan in patients with a history of kidney stones?. Am J Emerg Med.

[7] Hoppe H, Studer R, Kessler TM, Vock P, Studer UE, Thoeny HC (2006). Alternate or additional findings to stone disease on unenhanced computerized tomography for acute flank pain can impact management. J Urol.

[8] (2024). Choosing wisely. http://www.choosingwisely.org/societies/american-college-of-emergency-physicians.

[9] Catalano O, Nunziata A, Altei F, Siani A (2002). Suspected ureteral colic: primary helical CT versus selective helical CT after unenhanced radiography and sonography. AJR Am J Roentgenol.

[10] Pathan SA, Mitra B, Mirza S (2018). Emergency physician interpretation of point-of-care ultrasound for identifying and grading of hydronephrosis in renal colic compared with consensus interpretation by emergency radiologists. Acad Emerg Med.

[11] Raja AS, Pourjabbar S, Ip IK, Baugh CW, Sodickson AD, O'Leary M, Khorasani R (2019). Impact of a health information technology-enabled appropriate use criterion on utilization of emergency department CT for renal colic. AJR Am J Roentgenol.

[12] Leveridge M, D'Arcy FT, O'Kane D, Ischia JJ, Webb DR, Bolton DM, Lawrentschuk N (2016). Renal colic: current protocols for emergency presentations. Eur J Emerg Med.

[13] Schoenfeld EM, Pekow PS, Shieh MS, Scales CD Jr, Lagu T, Lindenauer PK (2017). The diagnosis and management of patients with renal colic across a sample of US hospitals: high CT utilization despite low rates of admission and inpatient urologic intervention. PLoS One.

[14] Watkins S, Bowra J, Sharma P, Holdgate A, Giles A, Campbell L (2007). Validation of emergency physician ultrasound in diagnosing hydronephrosis in ureteric colic. Emerg Med Australas.

[15] Malaki M (2014). The comparison of ultrasound and non-contrast helical computerized tomography for children nephrolithiasis detection. Urol Ann.

[16] Hastings RS, Powers RD (2011). Abdominal pain in the ED: a 35 year retrospective. Am J Emerg Med.

[17] Smith-Bindman R, Aubin C, Bailitz J (2014). Ultrasonography versus computed tomography for suspected nephrolithiasis. N Engl J Med.

[18] (2024). Xray risk. http://www.xrayrisk.com.

[19] Sartini M, Carbone A, Demartini A (2022). Overcrowding in emergency department: causes, consequences, and solutions-a narrative review. Healthcare (Basel).

